# Influence of reproductive history and exogenous hormone use on prevalence and frequency of circulating t(14;18)-positive cells in a population-based cross-sectional study

**DOI:** 10.1007/s10552-015-0525-4

**Published:** 2015-01-30

**Authors:** Kerstin Weitmann, Carsten Hirt, Sabine Schwarz, Charles Rabkin, Gottfried Dölken, Wolfgang Hoffmann

**Affiliations:** 1 Department Epidemiology of Health Care and Community Health, Institute for Community Medicine, University Medicine Greifswald, Ellernholzstr. 1-2, 17487 Greifswald, Germany; 2 Department of Haematology and Oncology, University Medicine Greifswald, Greifswald, Germany; 3 German Agency for Quality in Medicine ÄZQ, Berlin, Germany; 4 Division of Cancer Epidemiology and Genetics, National Cancer Institute, Bethesda, MD USA

**Keywords:** t(14;18) translocation, Healthy individuals, Reproductive history, Exogenous hormone use

## Abstract

**Purpose:**

The t(14;18) translocation might represent an intermediate step in the pathogenesis of follicular lymphoma (FL), one of the most common subtypes of non-Hodgkin lymphoma. Circulating t(14;18)-positive cells can also be detected in 30–60 % of healthy individuals at low frequencies. Some studies found a negative association between reproductive factors or use of menopausal hormone therapy (MHT) with FL. The objective of this study was to evaluate whether there is an association between number of frequencies, oral contraceptive (OC) use, menopausal status and MHT, and t(14;18) prevalence and frequency in a representative population analysis based on an epidemiologic study in the northeastern part of Germany.

**Methods:**

The analysis is based on results of buffy coat samples from 1,981 women of the Study of Health in Pomerania (SHIP-0) and data obtained in standardized face-to-face interviews. For prevalence, odds ratios (OR) and 95 % confidence intervals (CI) were calculated using unconditional logistic regression. Frequency data were analyzed using negative binomial regression. The multivariable models included age, number of pregnancies, menopausal status (premenopausal, natural, medical/surgical menopause), OC use and MHT as a measure for exogenous hormone exposure use.

**Results:**

We found no association between reproductive history and combined exogenous hormone use on the prevalence of circulating t(14;18)-positive cells. Modeling MHT and OC use separately in a sensitivity analysis, the MHT parameter yielded statistical significance [OR 1.37 (95 % CI 1.04;1.81)]. t(14;18) frequency was associated with use of OC [incidence rate ratio (IRR) for ever use 3.18 (95 % CI 1.54;6.54)], current use [IRR 3.86 (1.56;9.54)], >10 years use [IRR 3.93 (1.67;9.23)] and MHT [restricted to postmenopausal women; IRR 2.63 (95 % CI 1.01;6.85)] in bivariate age-adjusted analyses. In the multivariable model, medical/surgical menopause [IRR 2.46 (1.11;5.44)] and the category ever use of OC and MHT were statistically significant [IRR 2.41 (1.09;5.33)].

**Conclusions:**

Exogenous hormone use might be a risk factor for t(14;18) frequency rather than for t(14;18) prevalence. Further research on healthy individuals carrying a t(14;18) translocation and possible risk factors for malignant lymphoma is necessary to determine the additional molecular or immunological events that have to occur to develop FL.

**Electronic supplementary material:**

The online version of this article (doi:10.1007/s10552-015-0525-4) contains supplementary material, which is available to authorized users.

## Introduction

Non-Hodgkin lymphomas (NHL) comprise a heterogeneous group of lymphoid malignancies of different morphology and genetic characteristics [1]. Causes for common NHL subtypes remain largely unclear [2]. In epidemiologic studies, risk factors for NHL were often associated with the immune system [3], including HIV infection and immunosuppressive therapy following organ transplantation [2]. Female hormones like estrogen, progesterone, and prolactin modulate the immune system and influence the immune response. Estrogen decreases plasma levels of interleukin 6, which is a growth factor for intermediate- and high-grade NHL [4, 5]. During pregnancy, estrogen and progesterone levels rise [6, 7]. Hence, reproductive factors could explain the lower prevalence of NHL in females compared to males [8, 9].

The two most common subtypes of NHL are follicular lymphomas (FL) with about 20 % and diffuse large B cell lymphoma (DLBCL) with about 20–35 % of NHL cases [10–12]. In the USA, for both lymphoma subtypes, higher incidence rates have been reported in men than in women [FL: male–female incidence rate ratio (IRR) of 1.2 for Whites and Asians; IRR 1.6 for Blacks; DLBCL: male–female IRR of 1.6 for Whites; IRR 1.8 for Blacks; IRR 1.4 for Asians] [13]. However, systematic gender differences may be restricted to subgroups of the subtypes. Recent data from Europe show no sex differences or even a slightly reversed gender ratio of 0.9 for FL [14].

In both FL and DLBCL, high t(14;18) translocation prevalence rates have been detected (FL: 70–90 %, DLBCL: 30–50 %) [15–17]. The t(14;18) translocation involves the immunoglobulin heavy chain (IgH) locus on chromosome 14q32 and the BCL-2 gene on chromosome 18q21 [18]. The translocation results from an illegitimate V(D)J-recombination [19] and leads to a constitutive overexpression of the BCL-2 gene [20]. The resulting BCL-2 protein is involved in a deregulation of apoptosis [21]. Hence, the t(14;18) translocation might represent an intermediate step in the pathogenesis of both NHL subtypes [22, 23]. In animal studies, it was shown that estrogen treatment reduces early cµ+ pre-B cells and IgH gene rearrangements [24]. So estrogen might decrease the genesis of the t(14;18) translocation.

The t(14;18) translocation can also be detected in healthy individuals, as first described by Limpens et al. [25]. In Western countries, about 30–60 % of healthy persons are carrying this genetic translocation, whereas in Asia, lower prevalence rates were reported [26–28]. These results are in line with the low incidence of FL in Japan [29]. Similar to FL prevalence rates, higher prevalence rates of the t(14;18) translocation in males compared to females were observed in healthy individuals [26, 30–32].

Some studies reported negative association between FL and higher sex hormone levels [33–37]. Changes in estrogen hormone levels in women occur during and after pregnancy and menopause and can also be influenced exogenously by gender-specific drug use like oral contraceptives (OC) or menopausal hormone replacement therapy (MHT).

Particularly high t(14;18) frequencies in healthy individuals have been shown to be an early biomarker for FL [38]. Currently, it is unknown whether there is an association between sex-specific hormone levels and the prevalence of the t(14;18) translocation in healthy women. Therefore, the objective of our study was to evaluate a possible association between both the prevalence and the frequency of circulating t(14;18)-positive cells with number of pregnancies and menopausal status as well as gender-specific use of extraneous hormones such as OC and MHT in healthy women in a large representative population-based cross-sectional analysis.

## Materials and methods

### Study population

The Study of Health in Pomerania (SHIP-0) is a representative sample of the general population in the northeastern part of Germany. Details on the study design, recruitment and objectives have been published previously [39]. Briefly, in a two-stage design, a population sample of 7,008 men and women between 20 and 79 years was drawn randomly using German population registries. After exclusion of migrated or deceased persons, the net sample comprised 6,265 individuals. A total of 4,308 individuals (2,192 females, 2,116 males) participated (response proportion 68.8 %). Written informed consent was obtained from each participant. Aims of SHIP-0 are to estimate the prevalence of risk factors, subclinical disorders, and common clinical diseases as well as their complex associations.

Each participant underwent a comprehensive standardized medical examination. Data collection included a computer-aided personal face-to-face interview (CAPI) and a health- and risk factor-related questionnaire to be completed by the participants. Blood samples were drawn from the cubital vein according to standardized procedures. The SHIP-0 study was approved by the Ethics Committee of the University of Greifswald.

### Biosamples

The detection of the t(14;18) translocation in this study population was described previously [32]. The phenol–chloroform method was used to isolate DNA from peripheral blood buffy coat cells (nucleated cells, NC). DNA was stored at −20 °C. The t(14;18) MBR (major break-point region) of BCL2 was identified by using real-time quantitative PCR. Wild-type K-RAS gene (reference gene, two copies per genome) was determined as described previously [40]. For the t(14;18) assay, five replicates of 1 μg DNA were used. The t(14;18) MBR PCR assay had a sensitivity of one t(14;18)-positive cell in 5 × 10^5^ nucleated cells. A forward and reverse primer at a concentration of 400 nM, a TaqMan probe at a concentration of 200 nM, the standard TaqMan Universal PCR Master Mix (Applied Biosystems, Weiterstadt, Germany), and 1.0 μg DNA in a total volume of 50 µl were contained in the PCR mixture. The 2-min incubation at 50 °C to allow for cleavage by Uracil-N-Glycosylase was followed by incubation at 95 °C for 10 min to activate AmpliTaq Gold. Each PCR cycle included 15 s denaturation at 95 °C, and 1 min of combined annealing/extension at 61 °C. Dilution DNA of Karpas 422 cells was used to establish standard curves for the t(14;18) MBR PCR and for the K-RAS wild-type PCR. In all PCR experiments, appropriate positive and negative controls were included.

For analyzing the influence of reproductive history and exogenous hormone exposure on prevalence and frequency of circulating t(14;18)-positive cells, peripheral blood buffy coat samples from a total of 2,008 (91.6 %) women were available. A biosample was classified as t(14;18) MBR positive if ≥1 of the replicates was PCR positive. None of the t(14;18) negative samples met our a priori exclusion criterion of containing less 200,000 K-RAS genome equivalents. To obtain the t(14;18) frequency, the t(14;18) copies from all five replicates were summed up and afterward divided by the sum of tested cells (WT K-RAS copies: 2). The result was multiplied by 10^6^ to express the frequency as t(14;18)-positive cells per million NC.

### Assessment of reproductive history and exogenous hormone use

In the standardized computer-assisted personal interview (CAPI), reproductive history and menopausal status as well as gender-specific drug use including OC and MHT were solicited from all female participants in SHIP-0.

The number of pregnancies and the number of births were categorized as none, one, two, three, and four or more. Women who responded “yes” to ever having taken OC were classified as ever users. Women who responded “no” to the same question were classified as non-users. Based on the question concerning current use of OC, ever users were differentiated in current and past users; unknown current usage was categorized as unknown. Duration of OC use was categorized to >0 to <5, 5 to <10, and ≥10 years.

The determination of menopausal status was based on questions concerning presence of menstrual bleeding (yes/no), age at last period, and reason of cessation of periods (natural or medical/surgical). Women aged >40 years fulfilling the criteria of no menstrual bleeding for at least 1 year prior to the interview were categorized as being natural menopausal. Women reporting an induced cessation of menses were categorized as postmenopausal by medical/surgical reasons. Ever users of MHT were defined as women who answered “yes” to the following question: “If you are menopausal, did you ever take any hormones during the menopausal transition or thereafter, e.g., patches, tablets, injections?” Based on the reported duration of hormone use, the MHT users were classified in two categories (>0 to <5, ≥5 years). Approximate age at menopause was categorized in tertiles.

In the invitation letters, all participants were asked to bring their medication packages along and in the interview prescribed medication over the seven preceding days was solicited. On the basis of the provided medications, we were able to identify current MHT users. For an explorative analysis, we coded the medication according to the ATC (Anatomical Therapeutic Chemical classification system)-code subgroup GE03 “Sex hormones and modulators of the genital system.”

## Statistical analysis

Statistical analyses were performed with the software package STATA (Intercooled STATA, STATA/SE 10.1, StataCorp., Texas, USA). In the descriptive statistics, categorical data were expressed as percentages; continuous data were expressed as median and interquartile range (IQR). Two group comparisons were calculated using Chi-squared test (*χ* ^2^ test) for categorial data and Mann–Whitney-*U* test (MW-test) or Kruskal–Wallis test for continuous data. A *p* value <0.05 was considered statistically significant. All statistical tests were two-sided. Unconditional logistic regression was used to calculate adjusted odds ratio (OR) and 95 % confidence intervals (CI). For frequency data, negative binomial regression models were conducted and incidence rate ratio (IRR) calculated including only t(14;18) positive women. All models were weighted by the number of tested cells. The calculation of the likelihood ratio test is not possible with a simultaneous application of the weight option. Applying the likelihood ratio test by calculating un-weighted models yielded that our count data are over-dispersed. Since the negative binomial regression can be regarded as a generalization of Poisson regression and should be used in cases of over-dispersion, we decided to use the negative binomial regression method as the non-conservative method.

Prior results concern age and sex as influencing factors both to prevalence and frequency for t(14;18). Since only women were investigated, we included only age as potential confounder in the full models. The remaining model variables operationalize major determinants of hormonal status, e.g., number of pregnancies, menopausal status including type of menopause (medical/surgical, natural) and exogenous hormone exposure use (only OC, only MHT, OC and MHT vs. no exogenous hormone exposure). This operationalization was selected to reflect our hypotheses of a risk due to any exogenous female sex hormone rather than the effect of any specific source. In a sensitivity analysis, we provide corresponding results for a model in which ever use of OC and MHT were modeled as separate variables.

## Results

A total of 2,192 women (median: 49, IQR: 36–62 years) participated in the SHIP-0 cohort. Results of biosamples were available for 2,008 females. Altogether 17 women were excluded from this evaluation: four of these declined to be interviewed and 13 cases reported a history of cancer at the time of the interview. Missing data of variables included in the multivariable model yielded to an exclusion of 23 women.

One thousand nine hundred and sixty-eight women could be analyzed (89.8 % of the total study population) for the prevalence of the t(14;18) translocation. Descriptive characteristics of reproductive history (number of pregnancies, number of birth) and exogenous hormone use are presented in supplementary Table S1.

### Prevalence of t(14;18)

In 33.4 % (*N* = 657) of the women, at least one t(14;18) copy was detected per 10^6^ cells [prevalence of t(14;18)]. In older women, the prevalence was higher than in younger women. The median age of t(14;18) prevalent females was 53 years (IQR: 40–63), whereas the non-prevalent women had a median age of 46 years (IQR: 34–61) (MW-test: *p* < 0.001). For increasing 10-year age categories, we found a positive prevalence trend from 18.5 % in the age group 20–29 years up to 43.8 % in the age group 50–59 years, followed by a 3 % decline in the next age group 60–69 years down to 35.5 % in the last group of ≥70 years (Table 1).

**Table 1:** Association of t(14;18) prevalence with reproductive history and exogenous hormone use

Total	*N*	t(14;18)-negative	t(14;18)-positive (%)	Crude OR (95 % CI)	Age-adjusted OR (95 % CI)	Multivariable^a^ OR (95 % CI)
	1,968	1,311	657 (33.2)			
Age group						
20–29 years	270	220	50 (18.5)	1	–	1
30–39 years	381	271	110 (28.9)	1.78 (1.19;2.66)	–	1.72 (1.10;2.68)
40–49 years	370	257	113 (30.5)	2.01 (1.34;3.01)	–	1.80 (1.13;2.87)
50–59 years	368	207	161 (43.8)	3.54 (2.39;5.26)	–	2.93 (1.66;5.17)
60–69 years	334	198	136 (40.7)	3.05 (2.05;4.56)	–	2.57 (1.35;4.89)
≥70 years	245	158	87 (35.5)	2.52 (1.64;3.87)	–	2.25 (1.12;4.53)
Number of pregnancies						
Never pregnant (reference)	289	216	73 (25.3)	1	1	1
≥1	1,679	1,095	584 (34.8)	1.51 (1.13;2.04)	1.01 (0.72;1.41)	–
1	379	268	111 (29.3)	1.19 (0.82;1.71)	0.90 (0.61;1.33)	0.89 (0.60;1.32)
2	607	398	209 (34.4)	1.43 (1.03;1.99)	0.97 (0.67;1.40)	0.98 (0.67;1.42)
3	348	217	131 (37.6)	1.82 (1.27;2.60)	1.21 (0.80;1.81)	1.23 (0.81;1.85)
≥4	345	212	133 (38.6)	1.79 (1.25;2.57)	1.11 (0.74;1.66)	1.14 (0.75;1.72)
Number of births						
None (reference)	318	235	83 (26.1)	1	1	–
≥1	1,650	1,076	574 (34.8)	1.41 (1.06;1.87)	0.88 (0.64;1.22)	–
1	474	336	138 (29.1)	1.07 (0.76;1.50)	0.76 (0.53;1.09)	–
2	718	468	250 (34.8)	1.41 (1.03;1.92)	0.91 (0.64;1.30)	–
3	274	167	107 (39.1)	1.66 (1.15;2.39)	0.99 (0.65;1.49)	–
≥4	184	105	79 (42.9)	2.13 (1.42;3.19)	1.21 (0.77;1.91)	–
Use of OC						
Never (reference)	609	377	232 (38.1)	1	1	–
Ever	1,359	934	425 (31.3)	0.75 (0.61;0.93)	0.99 (0.74;1.31)	–
Past	676	479	197 (29.1)	0.83 (0.66;1.04)	0.97 (0.73;1.29)	–
Current	403	300	103 (25.6)	0.56 (0.42;0.75)	0.96 (0.65;1.44)	–
Past/current unknown	280	155	125 (44.6)	2.05 (0.85;4.90)	1.63 (0.65;4.09)	–
Total number of years of OC use^b^						
>0 to <5 years	352	247	105 (29.8)	0.67 (0.50;0.91)	0.89 (0.63;1.26)	–
5 to <10 years	335	237	98 (29.3)	0.70 (0.52;0.95)	1.21 (0.82;1.77)	–
≥10 years	662	442	220 (33.2)	0.83 (0.65;1.06)	1.02 (0.74;1.39)	–
Menopausal status						
Premenopausal (reference)	1,054	761	293 (27.8)	1	1	1
Postmenopausal	914	550	364 (39.8)	1.67 (1.37;2.05)	0.98 (0.66;1.45)	–
Type of menopause						
Natural	647	390	257 (39.7)	1.64 (1.32;2.05)	0.93 (0.61;1.42)	0.94 (0.61;1.43)
Medical/surgical	267	160	107 (40.1)	1.76 (1.31;2.36)	1.04 (0.68;1.58)	0.97 (0.63;1.48)
Use of MHT^c^						
Never (reference)	603	386	217 (36.0)	1	1	–
Ever	311	164	147 (47.3)	1.54 (1.15;2.07)	1.47 (1.09;2.00)	–
Total number of years of MHT use^b,c^						
>0 to <5 years	188	98	90 (47.9)	1.59 (1.12;2.25)	1.52 (1.07; 2.18)	–
≥5 years	119	65	54 (45.4)	1.42 (0.93;2.16)	1.34 (0.86;2.07)	–
Ever use of OC/MHT						
No OC/no MHT (reference)	467	302	165 (25.1)	1	1	1
No OC/MHT	142	745	67 (10.2)	1.53 (1.02;2.29)	1.37 (0.91;2.07)	1.42 (0.93;2.16)
OC/no MHT	1,110	796	314 (47.8)	0.72 (0.57;0.92)	0.96 (0.69;1.34)	0.94 (0.67;1.32)
OC/MHT	249	138	111 (16.9)	1.46 (1.05;2.03)	1.30 (0.89;1.90)	1.26 (0.86;1.86)

^a^Multivariable model including age, number of pregnancies, type of menopause, and exogenous hormone exposure use

^b^Do not sum up to 100 % due to missing data

^c^Restricted to menopausal women

OC, oral contraceptive; MHT, menopausal hormone therapy; OR, odds ratio (method: logistic regression); 95 % CI, 95 % confidence interval

Reproductive history and exogenous hormone use as potential risk factors for the prevalence of the t(14;18) translocation are described in Table 1. In our study group, 85.3 % of the women had at least one pregnancy. The maximum number of pregnancies in our sample was 12. Women reporting two pregnancies represent the largest group among the t(14;18) prevalent women. Among women without any pregnancy, 25.3 % were t(14;18) positive, whereas the prevalence of t(14;18) among women with at least one pregnancy was 34.8 % (*χ* ^2^ test: *p* = 0.001). The prevalence of the t(14;18) translocation increased with increasing number of pregnancies from 25.3 % in women with zero pregnancies up to 38.6 % in women with four or more pregnancies (Table 1). This positive association of the t(14;18) prevalence with number of pregnancies was observed in almost the same manner for an increasing number of births.

Ever users of oral contraceptives had a lower t(14;18) prevalence rate (31.3 %) than never users (38.1 %) (*χ* ^2^ test: *p* = 0.004). Among the postmenopausal women, a significant higher percentage was t(14;18) positive (39.8 %) compared to premenopausal women (27.8 %, *χ* ^2^ test: *p* < 0.001). t(14;18) prevalence did not differ for approximate age at menopause (<48 years: 36.6 %, 48 to >52 years: 44.4 %, ≥52 years: 39.7 %, *χ* ^2^ test: *p* = 0.14). The crude OR showed a positive association of the t(14;18) prevalence with the number of pregnancies, use of oral contraceptives and menopausal status, which are mostly influenced by age (Table 1).

Analyses of hormone replacement therapy (MHT) were restricted to postmenopausal women only. The t(14;18) translocation was detected in almost half of the women (47.3 %) who reported ever use of MHT. The prevalence of never users was significantly lower (36.0 %, *χ* ^2^ test: *p* = 0.002). In bivariate age-adjusted analyses, ever use of MHT compared to never use yielded a statistically significant OR of 1.47 (1.09;2.00) as well as MHT use >0 to <5 years [OR 1.52 (1.07;2.18)].

In the multivariable model, we included age, number of pregnancies, menopausal status including type of menopause and exogenous hormone use for oral contraceptive use and menopausal hormone replacement therapy. In this model, only age showed a significant association with t(14;18) prevalence with the highest OR of 2.93 in age group 50–59 years (Table 1).

The risk estimates of age, number of pregnancies and menopausal status in a logistic regression model including OC and MHT as separate ever/never variables revealed comparable OR with the model in which both types of use were operationalized in one variable. The OR for OC use in the sensitivity model was 0.92 (0.69;1.23) and very similar to the OC/no MHT [OR 0.94 (0.67;1.32)] category, the OR for MHT use was 1.37 which is slightly lower as the parameter estimate for the category no OC/MHT [OR 1.42 (0.93;2.16)], and reached statistical significance (95 % CI 1.04;1.81).

### Frequency of t(14;18)

Analyzing frequency data was restricted to t(14;18) positive women (33.2 %). Supplementary Table S2 provides median values of t(14;18) frequency per 10^6^ NC for reproductive factors and exogenous hormone use categorized by age. The median number of positive NCs for the whole sample was 3.86 per 10^6^ NC (IQR: 1.98-8.27) and increased with increasing age up to age group 60–69 (median: 4.75) followed by a small decrease in women aged ≥70 (median: 4.18). For the number of pregnancies, we did not observe statistically significant associations with t(14;18) frequency (Table 2). The age-adjusted parameter estimates up to three pregnancies were below one, only for women with more than three pregnancies the IRR was about 2.1-fold greater compared to never pregnant women that means t(14;18) frequency of women ≥4 pregnancies is over twice as high compared to never pregnant women. Ever use (IRR 3.18) as well as current (IRR 3.86) and past use (IRR 3.14) of OC showed a positive association with t(14;18) frequency as well as OC use ≥10 years in the bivariate age-adjusted models (Table 2). Median t(14;18) frequency did not depend on age at menopause [<48 years: 3.9 (IQR: 1.8-10.0); 48 to <52 years: 4.8 (IQR: 2.9-12.2); ≥52 years: 3.9 (IQR: 1.9-8.8), *p* = 0.06]. The age-adjusted IRR for women with medical/surgical menopause was increased [IRR 3.52 (1.25;9.89)]. In the models restricted to postmenopausal woman, a higher frequency of t(14;18) was observed among ever users of MHT [IRR 2.63 (1.01;6.85)] and among short-term users (>0 to <5 years) of MHT [IRR 4.56 (1.01;20.58)], but not among long-term users [≥5 years, IRR 1.08 (0.50;2.33)] (Table 2).

**Table 2:** Association of t(14;18) frequency with reproductive history and exogenous hormone use

	Number (*N* = 657)	Median (first; third quartile) of t(14;18) frequency among positives per 10^6^ NC	Crude IRR (95 % CI)	Age-adjusted IRR (95 % CI)	Multivariable^a^ IRR (95 % CI)
Age group					
20–29 years	50	3.15 (1.77;5.03)	1	–	1
30–39 years	110	3.72 (1.99;5.16)	0.94 (0.34;2.41)	–	1.08 (0.43;2.72)
40–49 years	113	3.13 (1.93;6.25)	1.23 (0.47;3.22)	–	1.43 (0.55;3.70)
50–59 years	161	3.97 (1.99;8.58)	0.97 (0.40;2.32)	–	1.15 (0.44;3.03)
60–69 years	136	4.75 (2.27;10.12)	6.35 (1.06;37.96)	–	3.67 (1.10;12.27)
≥70 years	87	4.18 (1.90;10.01)	1.15 (0.39;3.37)	–	2.19 (0.65;7.34)
Number of pregnancies					
Never pregnant (reference)	73	3.14 (1.79;5.63)	1	1	1
≥1	584	3.94 (1.99;8.38)	2.13 (0.66;6.91)	0.98 (0.51;1.90)	–
1	111	3.46 (1.83;6.80)	1.03 (0.47;2.26)	0.95 (0.44;2.05)	0.91 (0.43;1.94)
2	209	4.20 (2.11;9.08)	1.10 (0.54;2.24)	0.86 (0.44;1.68)	0.85 (0.44;1.64)
3	131	3.98 (2.43;7.99)	1.05 (0.48;2.28)	0.87 (0.40;1.93)	0.74 (0.35;1.55)
≥4	133	3.86 (1.92;9.11)	5.74 (1.00;33.11)	2.13 (0.74;6.14)	0.99 (0.48;2.08)
Number of births					
None (reference)	83	3.14 (1.76;5.63)	1	1	–
≥1	574	3.95 (1.99;8.41)	1.84 (0.55;6.14)	0.82 (0.41;1.65)	–
1	138	3.63 (1.93;8.16)	0.87 (0.42;1.82)	0.81 (0.38;1.73)	–
2	250	4.04 (2.03;9.01)	0.91 (0.45;1.84)	0.71 (0.35;1.45)	–
3	107	3.97 (2.44;7.37)	6.03 (1.03;35.26)	2.26 (0.68;7.53)	–
≥4	79	3.86 (1.76;9.13)	0.68 (0.32;1.45)	0.38 (0.18;0.80)	–
Use of OC					
Never (reference)	232	4.24 (2.12;9.19)	1	1	–
Ever	425	3.65 (1.92;6.91)	2.54 (0.74;8.68)	3.18 (1.54;6.54)	–
Past	197	2.96 (1.81;5.61)	3.01 (0.75;12.11)	3.14 (1.50;6.59)	–
Current	103	3.86 (2.22;6.25)	1.29 (0.67;2.48)	3.86 (1.56;9.54)	–
Past/current unknown	125	4.18 (1.83;9.13)	0.68 (0.32;1.45)	1.70 (0.74;3.90)	–
Total number of years of OC use^b^					
>0 to <5 years	105	4.05 (1.92;8.29)	1.00 (0.53;1.88)	2.05 (0.96;4.38)	–
5 to <10 years	98	2.79 (1.80;5.75)	0.93 (0.49;1.74)	2.05 (1.01;4.18)	–
≥10 years	220	3.82 (2.00;7.10)	3.99 (0.92;17.38)	3.93 (1.67;9.23)	–
Menopausal status					
Premenopausal (reference)	293	3.39 (1.91;5.33)	1	1	1
Postmenopausal	364	4.29 (2.05;9.49)	2.84 (0.80;10.18)	1.16 (0.71;1.90)	–
Type of menopause					
Natural	257	4.17 (2.03;8.99)	0.89 (0.61;1.29)	0.68 (0.38;1.24)	0.68 (0.38;1.23)
Medical/surgical	107	4.77 (2.10;11.27)	7.37 (1.48;36.58)	3.52 (1.25;9.89)	2.46 (1.11;5.44)
Use of MHT^c^					
Never (reference)	217	4.18 (2.05;8.46)	1	1	–
Ever	147	4.73 (2.09;12.05)	4.76 (0.89;25.49)	2.63 (1.01;6.85)	–
Total number of years of MHT use^b,c^					
>0 to <5 years	90	4.44 (2.17;12.23)	7.25 (1.20;43.77)	4.56 (1.01;20.58)	–
≥5 years	54	5.73 (1.99;11.93)	0.90 (0.45;1.80)	1.08 (0.50;2.33)	–
Ever use of OC/MHT					
No OC/no MHT (reference)	165	4.09 (2.00;8.34)	1	1	1
No OC/MHT	67	5.49 (2.60;12.23)	0.63 (0.35;1.12)	0.79 (0.44;1.43)	0.68 (0.40;1.16)
OC/no MHT	314	3.48 (1.92;6.04)	0.97 (0.54;1.73)	1.93 (0.95;3.93)	1.77 (0.94;3.33)
OC/MHT	111	3.91 (1.83;9.11)	5.96 (1.03;34.42)	4.55 (1.33;15.53)	2.41 (1.09;5.33)

Example of interpreting IRR. (1) age-adjusted IRR of postmenopausal women is 1.16; t(14;18) frequency is 16 % higher compared to premenopausal women, (2) age-adjusted IRR of women with 1 pregnancy is 0.95; t(14;18) frequency is 5 % less high compared to never pregnant women

^a^Multivarible model including age, number of pregnancies, type of menopause, and exogenous hormone exposure use

^b^Do not sum up to 100 % due to missing data

^c^Restricted to menopausal women

OC, oral contraceptive; MHT, menopausal hormone therapy; IRR, incidence rate ratio (method: negative binomial regression); 95 % CI, 95 % confidence interval; NC, nucleated cells

We did not observe a linear trend in the association between age and reproductive history and t(14;18) frequency in the multivariable model (Table 2). No statistically significant association was observed for number of pregnancies (IRR between 0.74 and 0.99). Natural menopause showed no association with t(14;18) frequency. The IRR for medical/surgical menopause, however, was significantly increased [IRR 2.46 (1.11;5.44)]. The multivariable model revealed no statistically significant IRR for the subgroup of women who had used only OC or MHT. For the subgroup of women who had used both IRR were increased 2.41 (1.09;5.33) (Table 2). In a sensitivity analysis, separate risk estimates for OC use and MHT were calculated. The IRR for OC use was statistically significant 2.29 (1.31;4.00), but not for MHT [IRR 1.02 (0.65;1.61)].

## Discussion

The biologic mechanisms for developing NHL are currently unclear. The t(14;18) translocation is the genetic hallmark for FL and DLBCL, two of the most common subtypes of NHL. The translocation can be found in 70–90 % of FL cases and in 30–50 % of DLBCL cases [15–17]. In Western countries, this mutation is detectable in about 30–60 % of healthy individuals [26–28]. Prevalence and frequency of t(14;18)-positive cells in healthy individuals are associated with known FL risk factors like increasing age and male sex [27, 28, 32, 41]. Roulland et al. [38] published very recently that the presence of t(14;18)-positive cells in healthy individuals is associated with an increased risk for FL and that these cells represent true lymphoma precursors which are clonally related to the clinically overt lymphoma that manifests at a later time point.

Epidemiologic observations link reproductive factors and gender-specific hormone use with risk of NHL [1, 9, 33]. Estrogen seems to be a protective factor for FL and DLBCL.

To the best of our knowledge, this is the first study investigating a possible association between circulating t(14;18)-positive cells and reproductive factors and exogenous hormone use in healthy females. In bivariate analyses, we found some positive associations between the t(14;18) prevalence and MHT use (ever vs. never, years of use). In the multivariable model, only age remains a statistically significant predictor showing a positive association with the t(14;18) prevalence. In a sensitivity analysis including OC use and MHT as separate variables in the model, an increased OR for MHT was observed. Restricting the multivariable model to postmenopausal women did not change the risk estimates significantly (data not shown).

Analyzing t(14;18) frequency in the multivariable model, the parameter estimates for age are less consistent and only age group 60–69 was associated with a significantly increased frequency (IRR 3.67). A positive association of medical/surgical menopause was observed (IRR 2.46). For ever use of both OC and MHT, the IRR of 2.41 was statistically significant. Restricting the multivariable model to postmenopausal women yielded comparable risk estimates (data not shown). A sensitivity analysis of OC use and MHT as separate variables in the model including all women yielded a statistically significant IRR for history of OC use. The separate parameter estimate for MHT was not statistically significant. Approximately three times as many women have ever taken OC compared to MHT (Table 1). Compared to MHT, OCs were taken on average at considerably younger ages. Based on these results, it could be speculated that exposure at younger ages may have a positive impact on t(14;18), while it does not affect prevalence.

Our multivariable analyses revealed no statistically significant parameter estimates for number of pregnancies, neither on prevalence nor on frequency. However, we observed decreased risks on t(14;18) frequency for women with at least one pregnancy. Several studies reported also a negative association between number of pregnancies and risk of DLBCL or FL [33–35, 42]. In line with this, two other studies showed an enhanced risk of NHL for null and low parity [43, 44].

Our results of the positive association between ever use of OC and t(14;18) frequency are consistent with Costas et al. [45] who reported slightly increased risks of FL (OR 1.49) and DLBCL (OR 1.28) with ever use of OC. Other studies reported inconsistent risks of NHL or its subtypes FL and DLBCL with use of OC [34, 46, 47]. This variation, however, might at least partly be attributable to low sample sizes.

Type of menopause was investigated by Morton et al. [9] in the National Institutes of Health-AARP Diet and Health Study cohort. The authors observed increased risks of women with surgical menopause versus women being natural menopausal (aged 50–54 years) for FL and DLBCL which is in line with our t(14;18) frequency results.

Inconclusive findings were published for MHT use. Among postmenopausal women, Mildon et al. [36] reported a decreased but statistically not significant risk of MHT users compared to non-users for FL (OR 0.6) and DLBCL (OR 0.7). Similar results were presented in a pooled analysis of the InterLymph case–control study for ever versus never MHT users with an OR of 0.82 for FL and an OR of 0.66 for DCBCL [37]. However, data from the California Teachers Study show elevated but not significant risks for ever versus never MHT use (FL: RR = 1.57; DLBCL: RR = 1.15) [47]. These results are in line with our bivariate age-adjusted findings restricted to postmenopausal women. Ever use of MHT compared to never use yielded a statistically significant OR of 1.47 in a prevalence analysis and a statistically significant IRR of 2.63 in a frequency analysis.

Cerhan et al. [48] compared current and former users with never users of MHT analyzing data from the Iowa Women’s Health Study cohort and found significantly increased risk estimates for FL (RR current: 3.3; RR former: 2.6), but no association for diffuse NHL. The SHIP interview did not differentiate between current use and former use of MHT. Based on the 7-day medication, we identified 13.7 % current MHT users among the 914 postmenopausal women with GE03 medication based on ATC classification. 44.8 % of these current users are carrying the t(14;18) translocation. The prevalence among women currently not taking any menopausal therapy is slightly lower (39.0 %) (*p* = 0.24). t(14;18) frequency of women currently applying MHT is not different compared to women who are not current users [4.47 (1.83;8.52) vs. 4.24 (2.10;10.11) per 10^6^ NC, *p* = 0.75].

Analyzing only the subgroup of current MHT users, we were able to differentiate estrogen, estrogen − progestin, and progestin users. We did not observe a difference of t(14;18) prevalence and frequency of women with current use of only estrogen versus estrogen + progestin as MHT [41 % vs. 46 %, *p* = 0.63; 3.58 (1.58;7.83) vs. 5.22 (2,31;8.61) per 10^6^ NC; *p* = 0.20]. The t(14;18) prevalence of MHT containing progestin only was 60 %, but this finding is limited to a small sample size (*N* = 5). Morton et al. reported similar risks for different types of MHT in FL and DLBCL patients [9]. Data from the California Teachers Study and the Iowa Women’s Health Study showed also increased risk estimates with duration of MHT use compared to never users, although sample sizes are often small [47, 48]. In bivariate age-adjusted analyses, we observed a statistically significantly increased risk for MHT use >0 to <5 years for prevalence (OR 1.52) as well as for frequency (IRR 4.56) but not for MHT use ≥5 years. We cannot completely exclude a possible bias due to a changing pattern of MHT use over time (e.g., dose and/or application pattern) or with respect to the application of different formulation/types of MHT.

Heterogeneous results in published risk estimates concerning NHL and hormone-associated factors in women might be attributable to a lack of differentiation of the NHL subtypes in many analyses. Only very few studies assessed possible risk factors separately for specific NHL subtypes. Other influencing factors include study setting, selection of confounding factors, small sample sizes, limited age range of participants, formulation of exogenous hormones, and duration of the application.

We observed a curvilinear association of age with both translocation prevalence and frequency. This observation is consistent with the age-specific incidence of FL. The median age at diagnosis for FL is about 65 years [49], which is comparable to the age with the highest t(14;18) prevalence and frequency in our study group. It is, however, unclear, why in older ages t(14;18) prevalence and frequency decline, while lymphoma incidence continues to increase.

Our analysis is based on the baseline examination of a population-based cohort, which is characterized by a high life-time prevalence of OC and MHT use [50]. The population-based sampling together with a high response proportion compared to other German cohorts [51] limit major selection bias and increases the external validity of these results. The study size of well-characterized women reduces statistical uncertainty. Information on reproductive history (number of pregnancies, parity) and hormone use (OC, MHT) was collected based on a standardized personal face-to-face interview (CAPI) conducted in designated study centers with each study participant. Reliability of self-reporting of reproductive factors and women health has been shown [52].

We were not able to evaluate the number of years after last birth, the age at menarche, the number of years of ovulation, and sex of the children born because the respective data were not collected. Furthermore, we did not consider hormonal influences of non-oral contraceptives like hormone injection or patches. In Germany, however, these methods play a minor role in contraception [53, 54], so we do not assume a considerable bias. Prescribed medication was solicited over the seven days preceding the interview. In the invitation letters, participants were asked to bring their medication packages along, and most did so. Data about calendar year or age of first/last OC medication, duration or pattern of application were not collected. Therefore, we were neither able to consider changes in OC formulation over the lifetime of women nor to perform a dose–response analysis.

In epidemiologic studies, no standardized definition for menopausal status exists [55, 56]. Due to (1) a variety of different definitions, (2) the complex processes of the transition from pre- to postmenopausal status, which can occur over several years or (3) a changing symptomology because of the use of MHT or hysterectomy, it is not trivial to characterize the menopausal status of women in population-based studies. We cannot exclude a potential misclassification of some of the women regarding their menopausal status. We used information on menstrual bleeding, which is difficult to evaluate in women who are taking MHT or had a hysterectomy or are in the perimenopausal status. Nevertheless, we only had 1.9 % of women aged ≥40 years with an uncertain menopausal status. Because of this small percentage, we assume no major bias, if any.

The t(14;18) translocation is the genetic hallmark for FL and DLBCL. The complex relationship by which exogenous hormone use like OC or MHT may lead to both NHL subtypes is not fully understood. It is known that sex hormones influence the B cell development [57]. Kane et al. indicate that estrogen at physiological levels suppresses the humoral response, whereas the exogenous estrogen from OC increases the humoral response. This leads to a reduction of IFN-γ and the enhancement of IL-6 and IL-10, which together may induce an increase of the number of B lymphocyte subpopulations and estrogen-induced expression of the BCL-2 gene which reduces apoptosis of B cells [42]. Both mechanisms could support the persistence of cells carrying the t(14;18) translocation over longer periods of time, which would explain the positive associations between t(14;18) frequency and use of OC and MHT observed in this study.

## Conclusion

To our knowledge, this is the first study analyzing t(14;18) prevalence and frequency data with regard to female reproductive history and exogenous hormone use in a population-based sample of healthy women.

In summary, we found a strong curvilinear trend of age on the prevalence of the t(14;18) translocation with a maximum among the 50–59 old and lower prevalence for the younger and older women, respectively. Our results suggest no relevant influence of number of pregnancies or type of menopause on the prevalence of the t(14;18) translocation.

For t(14;18) frequency, significant associations were observed with age-adjusted ever use of OC, and especially among current and long-term users. In the multivariable model, medical/surgical menopause and the category ever use of both OC and MHT showed significantly increased risk estimates.

Our results suggest that exogenous hormone use is a risk factor for t(14;18) frequency rather than for t(14;18) prevalence. In our study, some 80 % of all women prevalent for t(14;18) had only one clone. Only one woman had four different clones, which was the highest number of different clones observed. Hence exogenous hormone use might rather act on the expansion of existing clones than causing de novo mutations. Since it has been shown that the detection of t(14;18)-positive cells in healthy individuals represents a biomarker of increased FL risk [38], future research should aim at defining which individuals are at highest risk and which additional molecular or immunological events ultimately lead to the transformation of a t(14;18)-positive cell into a fully malignant lymphoma clone. A promising starting point might be a systematic prospective follow-up of healthy individuals carrying a t(14;18) translocation as a possible FL precursor in the SHIP-cohort. In a prospective approach, a variety of NHL risk factors can be monitored and their respective contribution toward developing clinical FL or DLBCL can be quantified.

## Electronic supplementary material

Below is the link to the electronic supplementary material.

Supplementary material 1 (DOCX 23 kb)

Supplementary material 2 (DOCX 31 kb)
