# Supplementary material for: Influence of reproductive history and exogenous hormone use on prevalence and frequency of circulating t(14;18)-positive cells in a population-based cross-sectional study
Source: Cancer Causes Control. 2015 Jan 30;26(3):455–65. doi: 10.1007/s10552-015-0525-4 (PMC4331597; doi:10.1007/s10552-015-0525-4)
Supplement: Supplementary file 1 — Supplementary material 1 (DOCX 23 kb) [file 10552_2015_525_MOESM1_ESM.docx]

Table S1 Descriptive statistic of reproductive history, exogenous hormone use and age (N=1968)

|  | 20-29 years | 30-39 years | 40-49 years | 50-59 years | 60-69 years | ≥70 years |
| --- | --- | --- | --- | --- | --- | --- |
| Total N | 270 | 381 | 370 | 368 | 334 | 245 |
| **Number of pregnancies** |  |  |  |  |  |  |
| never pregnant | 58.5% | 9.2% | 4.9% | 7.3% | 6.9% | 11.4% |
| ≥1 | 41.5% | 90.8% | 95.1% | 92.7% | 93.1% | 88.6% |
| 1 | 26.7% | 23.6% | 16.5% | 16.3% | 14.4% | 19.6% |
| 2 | 9.3% | 42.3% | 34.9% | 36.7% | 26.7% | 27.8% |
| 3 | 4.1% | 13.9% | 24.9% | 17.4% | 23.7% | 20.0% |
| ≥4 | 1.5% | 11.0% | 18.9% | 22.3% | 28.4% | 21.2% |
| **Number of births** |  |  |  |  |  |  |
| none | 64.4% | 11.3% | 5.4% | 7.9% | 6.9% | 11.8% |
| ≥1 | 35.6% | 88.7% | 94.6% | 92.1% | 93.1% | 88.2% |
| 1 | 24.8% | 31.0% | 24.9% | 22.3% | 18.0% | 22.5% |
| 2 | 10.0% | 47.0% | 49.7% | 44.6% | 28.4% | 28.2% |
| 3 | 0.0% | 8.9% | 14.1% | 14.7% | 26.1% | 19.2% |
| ≥4 | 0.7% | 1.8% | 6.0% | 10.6% | 20.7% | 18.4% |
| **Use of OC** |  |  |  |  |  |  |
| never | 4.8% | 7.9% | 8.7% | 29.4% | 56.0% | 97.6% |
| ever | 95.2% | 92.1% | 91.4% | 70.7% | 44.0% | 2.5% |
| past | 39.6% | 52.2% | 66.0% | 34.2% | - | - |
| current | 55.6% | 39.9% | 25.1% | 2.2% | - | - |
| past/current unknown | - | - | 0.3% | 34.2% | 44.0% | 2.5% |
| **Total number of years**  **of OC use**^1^ |  |  |  |  |  |  |
| >0-<5 years | 29.6% | 20.5% | 21.3% | 26.2% | 41.5% | 50.0% |
| 5-<10 years | 56.0% | 21.9% | 16.6% | 14.2% | 13.6% | 16.7% |
| ≥10 years | 14.0% | 57.5% | 61.5% | 58.8% | 42.2% | 16.7% |
| **Menopausal Status** |  |  |  |  |  |  |
| premenopausal | 100% | 98.7% | 84.9% | 25.3% | 0.3% | - |
| postmenopausal | - | 1.3% | 15.1% | 74.7% | 99.7% | 100% |
| **Type of menopause**^2^ |  |  |  |  |  |  |
| natural | - | - | 28.6% | 65.1% | 78.1% | 78.4% |
| surgical | - | 100% | 71.4% | 34.9% | 21.9% | 21.6% |
| **Use of MHT**^2^ |  |  |  |  |  |  |
| never | - | 100% | 69.6% | 50.2% | 63.1% | 86.1% |
| ever | - | - | 30.4% | 49.8% | 36.9% | 13.9% |
| **Total number of years**  **of MHT use**^1,2^ |  |  |  |  |  |  |
| >0-<5 years | - | - | 88.2% | 58.4% | 58.5% | 61.8% |
| ≥5 years | - | - | 11.8% | 40.9% | 40.7% | 32.4% |

^1^do not always sum up to 100% due to missing values; ^2^ restricted to menopausal women; OC: oral contraceptive; MHT: menopausal hormone therapy
