# Supplementary material for: Influence of reproductive history and exogenous hormone use on prevalence and frequency of circulating t(14;18)-positive cells in a population-based cross-sectional study
Source: Cancer Causes Control. 2015 Jan 30;26(3):455–65. doi: 10.1007/s10552-015-0525-4 (PMC4331597; doi:10.1007/s10552-015-0525-4)
Supplement: Supplementary file 2 — Supplementary material 2 (DOCX 31 kb) [file 10552_2015_525_MOESM2_ESM.docx]

Table S2: Median (1^st^ quartile;3^rd^ quartile) of t(14;18) frequency per 10^6^ NC by age for categories of reproductive history and exogenous hormone use restricted to t(14;18) positive women (N=657)

|  | **20-29 years** | | **30-39 years** | | **40-49 years** | | **50-59 years** | | **60-69 years** | | **≥70 years** | |
| --- | --- | --- | --- | --- | --- | --- | --- | --- | --- | --- | --- | --- |
|  | N | median  (1^st^ q.;3^rd^ q.) | N | median  (1^st^ q.;3^rd^ q.) | N | median  (1^st^ q.;3^rd^ q.) | N | median  (1^st^ q.;3^rd^ q.) | N | median  (1^st^ q.;3^rd^ q.) | N | median  (1^st^ q.;3^rd^ q.) |
| **Number of pregnancies** |  |  |  |  |  |  |  |  |  |  |  |  |
| never pregnant | 32 | 2.6 (1.7;4.8) | 7 | 2.5 (1.9;4.3) | 4 | 1.9 (1.4;3.3) | 11 | 6.5 (4.9;8.7) | 7 | 2.4 (1.9;4.7) | 12 | 3.5 (1.5;14.3) |
| ≥1 | 18 | 3.7 (1.9;5.1) | 103 | 3.7 (2.0;5.2) | 109 | 3.3 (2.0;6.3) | 150 | 3.7 (1.9;8.4) | 129 | 5.0 (2.5;10.2) | 75 | 4.4 (1.9;10.0) |
| 1 | 10 | 3.9 (1.9;4.4) | 27 | 4.5 (2.0;5.4) | 19 | 3.3 (1.7;12.7) | 23 | 2.7 (1.6;6.8) | 19 | 3.5 (1.8;7.2) | 13 | 3.7 (2.5;5.8) |
| 2 | 3 | 5.1 (2.2;11.3) | 46 | 2.9 (1.9;5.0) | 38 | 3.9 (2.2;9.8) | 61 | 4.1 (2.0;8.6) | 35 | 7.3 (3.3;18.2) | 26 | 4.5 (2.2;11.2) |
| 3 | 4 | 2.6 (1.7;3.5) | 22 | 4.0 (2.7;6.0) | 26 | 3.7 (2.4;6.2) | 33 | 3.8 (1.5;6.9) | 32 | 4.7 (3.1;9.5) | 14 | 5.5 (1.9;17.2) |
| ≥4 | 1 | 13.1 (-) | 8 | 3.5 (1.6;6.0) | 26 | 2.3 (1.5;3.7) | 33 | 4.1 (2.2;9.1) | 43 | 5.3 (1.9;12.4) | 22 | 4.3 (1.8;7.6) |
| **Number of births** |  |  |  |  |  |  |  |  |  |  |  |  |
| none | 35 | 2.5 (1.6;4.5) | 10 | 2.5 (1.9;4.3) | 5 | 2.4 (1.5;4.1) | 13 | 6.1 (4.5;8.6) | 7 | 2.4 (1.9;4.7) | 13 | 3.7 (1.7;7.7) |
| ≥1 | 15 | 4.1(2.2;9.7) | 100 | 3.7 (2.0;5.3) | 108 | 3.2 (2.0;6.3) | 148 | 3.7 (1.9;8.5) | 130 | 5.0 (2.5;10.2) | 74 | 4.3 (1.9;10.0) |
| 1 | 8 | 4.2 (2.8;7.0) | 33 | 4.6 (2.7;6.8) | 29 | 3.3 (1.7;6.2) | 31 | 2.7 (1.6;9.9) | 23 | 3.5 (1.8;8.3) | 14 | 3.4 (1.9;8.8) |
| 2 | 6 | 3.5 (2.2;5.1) | 52 | 2.9 (1.7;5.2) | 54 | 3.5 (2.1;8.4) | 77 | 4.0 (2.0;8.6) | 35 | 7.3 (3.8;18.2) | 26 | 4.8 (2.3;12.7) |
| 3 |  | - | 14 | 3.6 (2.7;4.0) | 15 | 3.0 (1.8;6.5) | 25 | 4.0 (2.3;6.6) | 39 | 4.8 (3.1;10.2) | 14 | 3.7 (1.9;10.0) |
| ≥4 | 1 | 13.1 (-) | 1 | 1.6(-) | 10 | 2.7 (1.5;3.7) | 15 | 3.4 (1.7;9.1) | 32 | 4.9 (1.8;11.8) | 20 | 4.5 (1.8;9.0) |
| **Use of OC** |  |  |  |  |  |  |  |  |  |  |  |  |
| never | 3 | 5.3 (1.8;13.1) | 10 | 3.7 (2.3;4.9) | 10 | 2.4 (1.5;3.9) | 48 | 3.5 (2.1;7.8) | 76 | 6.3 (2.8;12.3) | 85 | 4.2 (1.9;9.3) |
| ever | 47 | 3.1 (1.8;4.5) | 100 | 3.7 (2.0;5.2) | 103 | 3.3 (2.0;6.3) | 113 | 4.1 (1.9;10.0) | 60 | 4.0 (1.9;8.5) | 2 | 24.4 (3.9;44.8) |
| past | 26 | 2.8 (1.6;4.5) | 55 | 2.7 (1.8;4.5) | 68 | 2.8 (1.8;5.3) | 48 | 3.9 (2.0;8.2) |  | - |  | - |
| current | 21 | 3.1 (1.9;4.2) | 45 | 4.4 (2.5;6.8) | 34 | 3.9 (2.4;8.4) | 3 | 3.4 (1.8;34.3) |  | - |  | - |
| past/current unknown |  | - |  | - | 1 | 12.7(-) | 62 | 4.3 (1.8;14.7) | 60 | 4.0 (1.9;8.5) | 2 | 24.4 (3.9;44.8) |
| **total number of years of OC use**^1^ |  |  |  |  |  |  |  |  |  |  |  |  |
| >0-<5 years | 14 | 3.7 (2.2;5.1) | 17 | 4.1 (2.4;5.2) | 19 | 4.4 (1.7;9.9) | 30 | 3.0 (1.7;8.6) | 25 | 4.6 (2.1;8.6) |  | - |
| 5-<10 years | 29 | 2.7 (1.8;4.2) | 26 | 3.3 (1.6;7.2) | 16 | 2.5 (1.8;3.2) | 19 | 3.3 (2.3;6.5) | 7 | 3.5 (1.2;15.1) | 1 | 44.8 (-) |
| ≥10 years | 3 | 2.2 (1.4;3.7) | 57 | 3.6 (2.2;4.9) | 68 | 3.7 (2.1;7.3) | 64 | 4.6 (2.0;11.7) | 27 | 3.7 (1.8;8.5) | 1 | 3.9 (-) |
| **Menopausal Status** |  |  |  |  |  |  |  |  |  |  |  |  |
| premenopausal | 50 | 3.2 (1.8;5.0) | 109 | 3.7 (2.0;5.2) | 93 | 3.4 (2.0;6.5) | 41 | 3.0 (1.8;5.7) |  | - |  | - |
| postmenopausal |  | - | 1 | 4.3 (-) | 20 | 2.6 (1.7;4.4) | 120 | 4.1 (2.0;11.2) | 136 | 4.8 (2.3;10.1) | 87 | 4.2 (1.9;10.0) |
| **Type of menopause**^2^ |  |  |  |  |  |  |  |  |  |  |  |  |
| natural |  | - |  | - | 7 | 2.4 (1.5;5.8) | 79 | 3.4 (1.9;7.4) | 106 | 4.6 (2.2;9.0) | 65 | 4.4 (2.2;10.4) |
| surgical |  | - | 1 | 4.3 (-) | 13 | 2.8 (1.8;3.9) | 41 | 5.7 (2.5;12.0) | 30 | 7.8 (2.7;12.2) | 22 | 3.6 (1.5;7.7) |
| **Use of MHT**^1^ |  |  |  |  |  |  |  |  |  |  |  |  |
| never |  | - | 1 | 4.3 (-) | 13 | 2.4 (2.1;4.8) | 53 | 4.0 (2.0;7.2) | 73 | 4.5 (2.1;8.5) | 77 | 4.2 (1.9;10.0) |
| ever |  | - |  | - | 7 | 3.0 (1.3;3.9) | 67 | 4.3 (2.0;12.4) | 63 | 5.9 (2.7;12.2) | 10 | 4.3 (3.0;7.6) |
| **Total number of years**  **of MHT use**^1,2^ |  |  |  |  |  |  |  |  |  |  |  |  |
| >0-<5 years |  | - |  | - | 6 | 3.1 (1.7;3.9) | 38 | 3.2 (1.8;9.1) | 40 | 5.7 (3.3;13.6) | 6 | 7.4 (3.2;17.2) |
| ≥5 years |  | - |  | - | 1 | 0.8 (-) | 28 | 6.5 (2.6;14.2) | 22 | 7.2 (1.8;9.7) | 3 | 3.9 (1.3;4.6) |

^1^do not always sum up to 100% due to missing values; ^2^ restricted to menopausal women; 1^st^ q: 1^st^ quartile; 3^rd^ q: 3^rd^ quartile, OC: oral contraceptive; MHT: menopausal hormone therapy
